# Does emancipation devour its children? Beyond a stalled dialectic of emancipation

**DOI:** 10.1177/13684310211028382

**Published:** 2021-07-07

**Authors:** Margaret Haderer

**Affiliations:** Institute for Social Change and Sustainability (IGN), WU Vienna University of Economics and Business, Vienna, Austria

**Keywords:** Critical social theory, emancipation, Foucault, judgement, late modernity

## Abstract

Emancipation serves not only as a midwife for progressive agendas such as greater equality and sustainability but also as their gravedigger. This diagnosis underpins Ingolfur Blühdorn’s ‘dialectic of emancipation’, which depicts a dilemma but offers no perspective on how to deal with it. By drawing on Foucault, this article suggests conceiving of emancipation as a task moderns are confronted with *even if* a given emancipatory project has come to devour its children. Claiming autonomy from given social constellations is key to this task; key also is judging between legitimate and illegitimate claims to autonomy. In late modernity, the criteria for such judgement are no longer universally given. Instead of regarding the latter as entry into mere subjectivism (Blühdorn), this article presents judgement as a key political, ‘world building’-activity (Arendt), a *critical* social theory may join in, by not only observing the world but by also taking sides in it.

The modern concept and political rallying cry *emancipation* has come under pressure, especially those historically well-entrenched understandings of emancipation that have related the liberation from given social relations to a shift towards progressive ends, such as greater equality, democracy or sustainability. In the course of the upswing of right-wing politics in recent years, emancipation has been re-signified as liberation from legally established duties to others, such as asylum rights; from legally entrenched recognitions of the equality of all genders and sexes; and from science, such as climate science or – in the context of the recent Covid-19 pandemic – epidemiology, which are presented as threats to individual autonomy. Yet emancipation is also under pressure from another perspective, the perspective of global socioecological challenges, such as climate change. Without seeking to present emancipation and sustainability as a causally related zero-sum game, there is no doubt that the material standards of living which have become regarded as desirable standards for living an autonomous and self-determined life build on a social metabolism that consumes nature as if there were no planetary boundaries, no future generations and no other species. This is to say that one does not have to be a right-wing climate change denier nor an outspoken proponent of fossil-fuelled lives for ‘sustaining the unsustainable’ (Blühdorn, 2007) ‘at others’ expense’ (Lessenich, 2019).

In light of right-wing reconfigurations of and environmental pressures on common understandings of emancipation – reconfigurations and pressures this Special Issue engages with – it becomes clear that emancipation does not embody an ‘absolute value’ […] ‘fixed in eternity and hanging from the ceiling like herrings’ (Adorno, 2001, p. 175) but a continuously reconfigured and contested value, concept and political rallying cry (Butzlaff, in this Special Issue; Koselleck & Grass, 1998). Given current reconfigurations of and pressures on emancipation, the specific questions that drive this article are the following: How can emancipation, which has always been a key concept to critical social theory and its commitment to not only interpret the world but to also change it (Marx, 1978), still serve as a critical concept today? Is it still possible for emancipation to function as a norm and ideal that embodies not only claims to autonomy but also commitments to specific limits to autonomy, such as equality and sustainability, that is, as a norm and ideal of *constrained autonomy* in the service of inclusion and less destructive nature–society relations that is likely to trigger resonance in late modern societies as opposed to constituting a merely normative demand?

These questions are asked and answered against the backdrop of a social diagnosis that has sparked this Special Issue: Ingolfur Blühdorn’s diagnosis of a ‘dialectic of emancipation’. Blühdorn suggests that in late modernity, emancipation has become reconfigured in such a way that it has largely lost its critical and egalitarian thrust (Blühdorn, 2019, 2020a, 2020b; Blühdorn et al., 2020). Akin to Adorno and Horkheimer who have stated that the Enlightenment in the name of reason has not led to greater freedom but to humanity’s self-destruction (Horkheimer & Adorno, 2002), Blühdorn suggests that the Kantian Enlightenment idea(l) of emancipation has not led to greater autonomy *and* respect for the dignity of others but to self-regarding, distinction-seeking, singularizing, transgressive subjectivities (Blühdorn et al., 2020, pp. 120–132). These late modern subjectivities nourish ‘sustained unsustainability’ (Blühdorn et al., 2020), no less than right-wing authoritarianism (see Blühdorn and Lütjen in this Special Issue) and profess that the autonomous subject, once the ‘midwife’ of progressive emancipatory projects, has come to serve as their ‘gravedigger’ (Blühdorn, 2019, p. 158, 2020b, p. 48). Against the backdrop of this perspective, emancipation, it seems, *has* come to devour its children. Yet to what extent is this diagnosis convincing, a diagnosis that implies an all-encompassing sublation of hitherto understandings of emancipation? Equally important, what follows from this diagnosis? Does it signify the end of progressive emancipatory projects without redress, a ‘war of emancipation’ (Bauman, 2000, p. 51) without any exit in sight, the end of modernity to which not only the norm and ideal of autonomy has been key but also that of equality? These questions are the backdrop and starting point for this contribution.

While accepting the risk that a given project of emancipation may turn out to devour its children, and acknowledging that modernity has always meant reason *and* unreason (Adorno, 2008, p. 45), freedom *and* domination (Allen, 2017, p. 170), creation *and* destruction (Berman, 1988), this article questions the plausibility of the conceptual and argumentative moves that underpin Blühdorn’s diagnosis. The article argues that *even if* Blühdorn’s claim about emancipation *were* convincing, the claim that in late modernity it has boiled down to mere transgression, emancipation nevertheless remains the most promising ‘way out’ of such a constellation. Understanding emancipation as a ‘way out’ does not hinge on widely shared normative commitments but on a minimal sense of not being (fully) subject to and driven by given circumstances, imperatives and predicaments, that is, a minimal sense of and capability to autonomy. That humans do have such a sense and capability is a basic assumption of modernity. Against this backdrop, emancipation constitutes a task moderns are confronted with as long as they conceive of themselves as moderns (see also Foucault, 2003b). Conceiving of emancipation as a (lingering) task, this article argues further, implies two aspects: claiming autonomy from social pre-givens and imperatives (which may include the imperative of transgression); and judgement with a view to the scope and limits of a given take on autonomy. This is what makes emancipation, at least from a conceptual history perspective, distinct from other concepts, such as liberation or transgression (Koselleck & Grass, 1998).

For Kant, the criteria for such judgement were transcendentally given and accessible by reason, criteria of which the categorical imperative is a case in point (Kant, 2006b). In late modernity, beliefs in rationally accessible, transcendentally given criteria for judgement have clearly crumbled. Yet, while some may regard the disintegration of transcendentally given, rational, universal or at least widely shared standards for delimiting claims to autonomy as a point of entry into people becoming judges in their own affairs (Blühdorn and Butzlaff, in this Special Issue), this article offers a different perspective, one informed by an Arendtian take on judgement (Arendt, 1993a; Zerilli, 2015, 2016). Having *only* contextual, socially embedded and historically rooted criteria for judging between legitimate and illegitimate claims to autonomy at hand is, as Arendt and Linda Zerilli, who draws on Arendt, argue, not forcibly a point of entry into mere subjectivism, as Blühdorn implies and Bauman insinuates by warning of ‘a war of emancipation’ (2000, p. 51). It may also be viewed quite differently: as a point of entry into the realm of actual (and conflictual) politics. Limit-setting through judgement, this article suggests, is a profoundly political activity, one that appeals to the consent of others, fully aware that some will agree and others disagree (Arendt, 1993a). In addition, limit-setting does not occur in a void but operates with and against criteria that have emerged in and through past social and political struggles for emancipation, that is, *political* criteria for limit-setting that may be regarded as legacies of modernity. These legacies, which are pluralistic in nature, constitute an inheritance (Allen, 2017, p. 165) that is certainly subject to reconfigurations due to processes of modernization but hardly subject to an all-encompassing hollowing out, as Blühdorn suggests.

The structure of the article is as follows: in the second section, I briefly recount key elements of Ingolfur Blühdorn’s ‘dialectic of emancipation’; the third section takes issue with three of the diagnosis’ conceptual and argumentative operations: first, the declaration of death of an *ideal* (autonomy with limits) in light of non-corresponding *social* norms (autonomy from limits), which omits the epistemic difference between norms and ideals; second, its lack of differentiation between modernization-induced and politically delimited norms of autonomy and emancipation; and third, its employment of dialectic, which seems to stall at negation and conjecture. The fourth section takes a step away from reconfigurations of dialectics and argues, more generally, that even if a given take on emancipation were to undercut its initial goals and intents, this would hardly mean the end of emancipation as a critical vantage point or normative horizon, but rather a call to critical reorientation and political repositioning through judgement. It does so by drawing on Foucault’s take on the very meaning of the Enlightenment, namely as a ‘way out’ of social pre-givens and by combining it with an Arendtian outlook on judgement: a context-dependent, political activity which may appeal to or go beyond given legacies of emancipation. In the conclusion, implications for social theory will be discussed.

## Ingolfur Blühdorn’s dialectic of emancipation

In his writings, including his contribution to this Special Issue, the social theorist and environmental scholar Ingolfur Blühdorn expresses profound doubt as to the transformative potential of emancipation as a way of shifting late modern democracies towards greater equality or sustainability (Blühdorn, 2019, 2020a, 2020b; Blühdorn et al., 2020). In fact, he argues that the norms and ideals of autonomy and the autonomous subject, once the ‘midwife’ of progressive emancipatory agendas, seem to have become these agenda’s ‘gravedigger’ (Blühdorn, 2019, p. 158, 2020b, p. 48). In late modern societies, Blühdorn notes, there is a ‘conspicuous absence of any major ‘‘revolutionary potential’’ (Blühdorn, 2020a, p. 394) for a shift towards more egalitarian or sustainable social relations. Even worse, there is a conspicuous *presence* of a lack of desire for such a shift, embodied by recent surges of ‘autocratic authoritarianism’ (Blühdorn, in this Special Issue). The decline of progressive emancipatory agendas, as he proposes, is not sufficiently explained by the ‘oppression’ of the ‘autonomous subject’, its subjection to capitalist logics that stand in the way of more authentic and fulfilling forms of ‘self-determination’ and ‘self-realization’ as critical social theorists commonly suggest (Blühdorn, 2020a, pp. 394–395). Instead, the absence of progressive ‘revolutionary potentials’ may itself be an indication that the idea(l), ‘the autonomous subject’, may have become ‘exhausted’ or ‘reframed in such a way that it no longer conflicts with the factual realities’ (Blühdorn, 2020a, pp. 394–395). In fact, ‘the autonomous subject’ may even feel at home in current ‘autocratic authoritarian’ reconfigurations of these realities (Blühdorn, in this Special Issue). How come?

In order to explain this social constellation, Blühdorn turns to processes and theories of modernization, especially theories on individualization (Bauman, 2000; Beck & Beck-Gernsheim, 2002; Giddens, 1991; Reckwitz, 2020; Sennett, 2006), which he considers as key to making sense of current reconfigurations of norms and ideals of autonomy and the autonomous subject. The theories suggest that in late modernity, in which hitherto ‘solids’ (such as communities, family ties, traditions, and social and economic securities) have become ‘liquefied’ at a rapid pace (Bauman, 2000), a process that turns ‘the autonomous subject’ into an increasingly self-producing one. As Beck and Beck-Gernsheim put it, ‘the individual is becoming the basic unit of social reproduction’ (2002, p. xxii), which renders categories, such as class, gender, the family or the neighbourhood into ‘zombie categories’, that is, into categories that may be alive sociologically, but that are dead empirically (2002, p. 203). For social theory to be up to speed with late modern developments, sociology needs to turn its attention to the liberating but also burdensome dimensions of making ‘day-to-day decisions about how to behave, what to wear and what to eat’ (Giddens, 1991, p. 14), a task individuals are confronted with in a social context that is shaped by acceleration (Bauman, 2000; Rosa, 2013), demands for flexibility (Sennett, 2006) and pressures towards cultural valorization and distinction, which Reckwitz refers to as ‘singularization’ (Reckwitz, 2020). Acknowledging that late modern processes of individualization are embedded within ‘globalized market-economies’ (Blühdorn, in this Special Issue), Blühdorn argues that ‘actually existing’ autonomy – individual self-production – is a type of autonomy that bears greater resemblance to a (neoliberal) ‘survival strategy’ than to the becoming real of an Enlightenment ideal (Blühdorn, 2009, pp. 24–25).

In light of these processes of modernization and their production of new forms and norms of autonomy and subjectivity, Blühdorn claims that any social theory that holds fast to the ideal of autonomy and emancipation in its name – a core ideal to progressive political agendas – *without* also carefully engaging with *factually occurring* reconfigurations of autonomy runs the risk of collapsing into mere normative theory (Blühdorn, in this Special Issue). Especially critical social theory, to which the ideal of the autonomous subject is central in one form or another, is hard pressed to reposition and rethink this ideal against the backdrop of actually existing social constellations (Blühdorn, in this Special Issue) – a repositioning critical theorists are clearly aware of. As Honneth puts it: ‘Without a *realistic* [emphasis added] concept of the “interest in emancipation,” which supposes an incorruptible core of rational susceptibility on the part of the subjects for the purpose of criticism, this theoretical project has no future’ (Honneth, 2008, pp. 807–808). Blühdorn himself claims to deliver a *realistic* concept of the ‘interest in emancipation’ in late modern democracies, namely the emancipation from Enlightenment ideals of emancipation, from, what he calls ‘first order emancipation’ (Blühdorn, 2020a, p. 400).

For Blühdorn, Kant’s take on autonomy and emancipation, the exit from self-imposed immaturity (Kant, 2006a), is *the* paradigmatic case of ‘first order emancipation’ (Blühdorn, 2020a, p. 400). The core feature of first-order emancipation is that it implies not only liberation from (given or self-imposed) limits embodied by God, Nature and the King but also the subjection to new limits: the limits of reason. In the field of morality, this means subjection to the categorical imperative, which suggests acting ‘only in accordance with that maxim through which you can at the same time will that it becomes a universal law’ (Kant, 2006b, pp. 4, 421) – a law by which the autonomous subject secures her own autonomy by also treating others as ends in themselves, that is, as autonomous. Yet in late modernity, Blühdorn argues, this rational, limit-setting dimension of emancipation that has pertained not only to Kantian moral philosophy but also to the workings of liberal democracies where it has re-emerged as *bürgerliche Vernunft* [bourgeois reason], maturity and public reason (Blühdorn et al., 2020, pp. 120–132) seems to have evaporated. Emancipation understood as the actualization of autonomy has become ‘mere liberation’, the transgression of any limits except the ones useful to one’s own self-production and optimization. Thus, whereas Kant depicted the Enlightenment as ‘emergence from self-imposed immaturity’ (Kant, 2006a), Blühdorn describes emancipation in late modernity as ‘the emergence of contemporary individuals and societies from their self-imposed maturity, that is, ‘‘second order emancipation’’ (Blühdorn, 2019, p. 157, 2020a, p. 397, 2020b, p. 45). The distinguishing features of the latter are that this type of emancipation ‘cannot accept any boundaries, is inherently transgressive and, invariably, questions (politicizes) its own foundations, which had remained unchallenged (pre-political) so far’ (Blühdorn, 2020a, p. 397). It is against this backdrop that Blühdorn suggests that the very norm that once gave birth to emancipation and progressive political agendas, such as democracy, greater equality and sustainability, has metamorphosed into the gravedigger of such agendas, which Blühdorn depicts as a ‘dialectic of emancipation’ (Blühdorn, 2019, 2020a, 2020b).

Yet to what extent is this account of reconfigurations of autonomy and emancipation convincing? Has emancipation really boiled down to mere transgression? Has, as Blühdorn claims, the ‘agenda of autonomy and emancipation’ indeed stopped animating shifts towards greater equality and sustainability or other progressive agendas in late modernity? The stakes are clearly high, not only against the backdrop of progressive political agendas (which one may or may not share). They are high because leaving the ‘agenda of autonomy and emancipation’ behind, hardly means less than leaving modernity behind, one of whose hallmarks is to conceive of human beings as autonomous and capable of distancing themselves, at least minimally, from given circumstances in order to reflect critically on them. Yet how far emancipatory agendas must give way to Blühdorn’s analysis obviously depends on the plausibility of the diagnosis, which I will now critically investigate.

## From midwife to gravedigger? Three critiques

### Regulative ideals, social norms: Or why the real does not constitute a death blow to the ideal

One central intent of Blühdorn’s ‘dialectic of emancipation’ (Blühdorn, 2019, 2020a, 2020b) is to capture a cultural shift, a shift in social norms to which, as he argues, critical social theory tends to pay too little attention and makes normative claims instead (Blühdorn, in this Special Issue). While holding fast to the regulative ideals of autonomy and emancipation without paying attention to shifting social relations may indeed imply that social theory, especially if it understands itself as *critical* theory, is out of sync instead of in touch with given social relations, Blühdorn’s own diagnosis runs the reverse risk: disbanding normative ideals altogether. By suggesting that late modern social norms have dug a grave for ‘classical understandings’ (Blühdorn, in this Special Issue) of emancipation such as the Kantian one, Blühdorn turns a blind eye to the *epistemic difference* between social norms and regulative ideals. Changing norms of autonomy and emancipation due to processes of modernization may certainly usher in a lack of desire for or distance from a given regulative ideal (e.g. autonomy constrained by reason). It is unclear, however, why such a lack of desire would undermine the ideal itself, whose validity hinges on being – in principle – right (or desirable) instead of real. The relationship between social norms and ideals, which Blühdorn presents as a relationship shaped by reversal, is equally likely to be one that produces tensions, ambivalences, and contradictions and, relatedly, social change, given the epistemic difference between norms and ideals.

To illustrate this point, going back to Kant may help. Kant conceived of autonomy constrained by reason in the form of the categorical imperative as a regulative ideal (*what ought to be*) instead of a social norm (*what is*). The categorical imperative suggests that human beings *ought to* act as if we they were members of the ‘kingdom of ends’, that is, members of an ideal order in which human beings treat themselves and others as autonomous beings (Kant, 2006b, pp. 4, 439), *regardless* of how Hobbesian others may be in relation to us. For Kant, acting according to the categorical imperative is an *unconditional* demand of reason (Kant, 2006b, pp. 4, 416). This demand of reason remains unconditional *even if* subjects face barriers to acting on it (the Hobbesian behaviour of others) and *even if* subjects come to rid themselves of this demand (Blühdorn’s diagnosis with view to late modern subjects). Thus, from a Kantian perspective, the social fact that the categorical imperative is not acted upon or even actively transgressed at a given point in time is clearly problematic and deplorable, but non-action on an ideal does not suggest that the ideal itself is invalid (see also Dobson, in this Special Issue).

The point here, to be clear, is neither to defend Kant and his specific take on setting limits to autonomy nor to defend purely normative theory. The point is that regulative ideals (which are not only the product of abstract reasoning but also that of social struggles) may not simply pale into insignificance, when they are – for whatever reason – not acted upon. The twentieth century, one may argue, was replete with liberations from Kantian (and, more generally, liberal) ideals of emancipation. Totalitarianism is a case in point, which Arendt had conceived as the *definitive* political event in the twentieth century, because it signified the collapse of all inherited criteria for judgement (Arendt, 1992). Yet in the same century, Kantian (regulative) ideals resurfaced prominently in the context of post-WWII liberal constitutionalism and international law. After having been insignificant for years, these ideals were – for good or worse (on the ambivalence of these regulative ideals, see Pitts, 2018) – deemed again fit for the normative guidance of post-WWII constitutionalism and international law. Against this backdrop, it may be premature to declare an ideal as exhausted in light of a non-corresponding social reality. In addition, such a declaration of death may also be read as indifference to the *epistemic* nature of normative reason: its deliberate distance from the real – one precondition for critique.

### Emancipation as an effect of modernization, emancipation as politics

Being a subject in late modern consumer societies means being socialized into a world of the continuously accelerating dissolution of ‘solids’ and hitherto securities (Bauman, 2000); individualizing forms of government (Beck & Beck-Gernsheim, 2002; Giddens, 1991), pressures to produce and optimize oneself (Sennett, 2006) and singularization (Reckwitz, 2020), the search for uniqueness and distinction. While processes of modernization and their reconfiguration of subjectivity are observable facts in a ‘globalized market-society’ (Blühdorn, in this Special Issue), it is less clear to what extent these processes and reconfigurations embody forms of autonomy and emancipation. Answering this question hinges on reconstructions of what the subjects involved *experience* (Honneth, 2002, p. 142) – reconstructions that, in the case of Beck, Giddens, Bauman, and Sennett, and also Blühdorn deliver an ambivalent picture: subjects experience freedoms *as well as* new burdens, risks and constraints. Yet answering this question also depends on a more foundational question: To what extent is one ready to let go of conceptual history, which is always, but, regarding emancipation, *especially* steeped in *social* and *political* history (Koselleck & Grass, 1998) – and solely focus on modernization-induced norms and meanings instead? From a conceptual and social history perspective, one may clearly be hesitant to readily conceive of individualization as a form of emancipation or of (neo-liberal) self-production and flexibilization as a form of autonomy.

Certainly, one may regard emancipation as an ‘essentially contested concept’ (Gallie, 1955) and subsume all sorts of meanings under it without further looking into *contexts* of meaning-making and their respective implications. Letting *any* claim to or experience of autonomy or emancipation pass as autonomy or emancipation obviously bears a risk: the risk of dissolving concepts into analytical indistinctiveness, which clearly undermines their critical thrust. Although emancipation does certainly not constitute a value fixed in eternity, it is safe to say that in modernity, emancipation comes with *specific* normative horizons, such as claims to self-determination, democracy, political and social participation, the rule of law, equality and more sustainable nature–society relations. Understanding emancipation as a profoundly *political* concept, an understanding which the history of the concept clearly invites (Koselleck & Grass, 1998), provides a critical vantage point on reconfigurations of emancipation through processes of modernization. Invitations to conceive of ‘neoliberal survival strategies’ (Blühdorn, 2009, pp. 24–25) as emancipation may be – in light of the concepts *political* history – also declined.

In addition, understanding emancipation as a political concept that comes with various normative horizons and that has left various social and political legacies – inclusionary *as well as* exclusionary ones, empowering *as well as* subjecting ones – may prevent us from presenting the history of emancipation as the alternation of (only) two orders: a Kantian (liberal) order that sets (rational) limits to autonomy (first-order emancipation) and a late modern order that transgresses any limits (second-order emancipation). The social, political and conceptual history of emancipation has left an inheritance that clearly exceeds Blühdorn’s two orders and even presents the orders themselves as more ambivalent than Blühdorn depicts them. Kant’s individual autonomy-focused, rational take on emancipation was challenged by Hegel’s intersubjective, Marx’s materialist, Herder’s romantic and nationalist or Nietzsche’s transgressive, self-producing take on autonomy and emancipation. In addition, constraining autonomy by (universal) reason did itself not only foster inclusion but also exclusion, a devaluating ‘politics of difference’, and the subjection of non-human nature. Depicting women as *perpetually* immature in civil matters plays a central role in Kant’s take on moral agency, which *cannot* be explained away by suggesting that Kant was a child of his time (see Marwah, 2013). In addition, Kant was a thinker of racial difference, not only in a pluralist sense but also in an evaluative sense. Committed to a ‘universal humanity’, he also stated that humanity is in its greatest perfection in the White race (see Vial, 2016; Marwah, 2019). Last but not least, Kant’s conception of the realm of nature (including non-human nature) as ‘the other’ of the realm of freedom has been problematized by greens for decades and is re-problematized in this Special Issue (see Dobson). Thus, even in the context of what Blühdorn calls first-order emancipation, autonomy also implied (justifications for) living at the expense of others, including non-human nature. The point here is not to dismiss the Kantian take (or any other rationalist, Universalist take) on autonomy *tout court*. The point is to invite a differentiated as opposed to nostalgic look at what a given take on autonomy involves conceptually and historically. Once such a look has been taken, one may embark on answering the question of how one may relate to a given take, history and legacy of emancipation and which elements of a given take, history and legacy one may reclaim or discard in the present.

### A stalled, conjectural dialectic?

Commenting on Adorno and Horkheimer’s diagnosis of a *Dialectic of Enlightenment,* Habermas once remarked that the analysis lends itself to being read as a story of decline and fall rather than as a dialectic, that is, as a *Verfallsgeschichte*, which tends to be ‘insensitive to the highly ambivalent content of cultural and social modernity’ (Habermas, 1987, p. 338). Leaving aside the question of whether Habermas was right with a view to Adorno and Horkheimer, one may argue that Blühdorn’s ‘dialectic of emancipation’ runs a similar risk. By conceiving of norms and ideals as standing in a relation of negation (from midwife to gravedigger), it cancels the claimed dialectic between first- and second-order emancipation. Dialectical thinking typically embodies three elements: a thesis (which is shaped by an incompleteness), an antithesis (which makes the incompleteness of a given thesis visible and negates *as well as* preserves parts of the thesis) and a synthesis (which embodies elements of the old as well as the antithesis and thereby embodies something new). This type of dialectic is usually referred to as Hegelian although Hegel himself scarcely employed these terms (Maybee, 2020).

The phrase from midwife to gravedigger clearly speaks the language of negation but omits the language of preserving. In Blühdorn, the ‘new’, second-order emancipation seems to be negating ‘the old’, first-order emancipation. While there is nothing wrong per se with stating a relationship of negation, the latter embodies a dialectic that has stalled half way and that does away with ambivalences and contradictions, which are in fact a hallmark of dialectical thinking. In addition, for a relationship to qualify as a dialectical one, establishing an actual as opposed to a conjectural relation between thesis and antithesis is key. There is, to illustrate this point, an actual relationship between master and slave: without slave, there is no master and vice versa. Yet, one may ask, what is the actual relationship between the Kantian autonomous subject (Blühdorn’s thesis) and processes of (late modern) modernization (Blühdorn’s antithesis)? Why did the one evoke the other as opposed to contingently coexist with it? While there may be a dialectical relationship (the *liberal* nature of both may embody the missing link), the relationship requires establishing, akin to how Dobson and Arias-Maldonado (in this Special Issue) establish a relationship between the Kantian norm of autonomy (which implies freedom from nature, including non-human nature) and the Anthropocene (which may be read as a problematization of this freedom).

Not quite convinced by the argument that there *is* a dialectic of emancipation that pushes ‘truly critical thinking’ about late modern societies towards leaving ‘the agenda of autonomy and emancipation’ wholesale behind, in what follows, I take a step aside from dialectical thinking towards a thinking that presents emancipation as a *lingering task* moderns are confronted with *qua* being moderns. The ensuing analysis focuses less on given reconfigurations of emancipation than on the question of how one may relate to a given take on emancipation that could be thought to have ‘gone wrong’, such as the transgression of any limit, in the absence of rational, universal or widely shared standards of adjudicating the scope and limits of autonomy and emancipation in its name. Claiming autonomy from a given configuration of emancipation is the response to the first challenge (the question of emancipation ‘gone wrong’). Accepting and asserting the context-specific, historically informed, contingent nature of limit-setting through *political judgement* is the response to the second challenge (the post-foundational nature of late modernity).

## Towards conceiving of emancipation as a (lingering) task

Emancipation clearly has the capacity to devour its own children, as Blühdorn’s diagnosis suggests. Yet even if we see a specific *project* of emancipation undercutting itself – a project that promised ‘perpetual peace’ (Kant), the realization of one’s ‘species being’ (Marx) or ‘true’ autonomy, equality or sustainability – one may argue that *the task* of emancipation remains, even if a given emancipatory project has disastrously failed, not been realized for too long, changed its meaning or purpose, or even turned into its opposite. Especially given the logic of cancellation that underpins Blühdorn’s account of the relationship between norms and ideals and his emphasis on the moment of negation in his account of dialectics, Blühdorn’s diagnosis *does* open the door to the possibility of leaving the normative inheritance of modernity behind, to which autonomy and emancipation certainly belong. While some discard such a ready door-opening as reactionary (Neckel, 2020), one may simply accept it as an invitation to consider and engage with the possibility that social change may take us beyond autonomy and emancipation at some point. But has it already?

While accepting that modernity, akin to every other period in history, is unlikely to last forever, it is hard to deny that modernity has left manifold legacies – a rich and ambivalent inheritance – that is still operative in the present. That we still speak of norms and ideals of autonomy and emancipation, including their promises, pitfalls and reconfigurations – as we do in this Special Issue – is but one case in point. When conceiving of modernity as an ‘inheritance’ (Derrida, 1994), the question emerges of how to relate to it. One way of relating to it is to accept it as a lingering *task* that implies affirming an inheritance ‘in and through’ its radical transformation’ (Allen, 2017, p. 165; Derrida, 1994). Relating to emancipation in this way constitutes one way out of the stalemate Blühdorn’s ‘dialectic of emancipation’ may create in respect of progressive emancipatory agendas.

While Derrida (1944) and Allen (2017, p. 165) map a way of how late moderns may relate to the inheritance of modernity, Foucault suggests conceiving of emancipation itself primarily as a ‘task and obligation’ (Foucault, 1984, p. 35). In his engagement with Kant, Foucault is not searching for an authoritative, philosophical ‘*project* of emancipation’ as Blühdorn does (Blühdorn, in this Special Issue), but for how Kant makes sense of his own historical conjuncture, the Enlightenment and the challenges that come with it and with modernity more generally. Kant, Foucault argues, in regard to Kant’s answer to the question of what is Enlightenment (Kant, 2006a), is not seeking to understand the Enlightenment ‘on the basis of a totality or of a future achievement’, such as ‘rational subjectivity’ or ‘perpetual peace’ – a project. Instead, as Foucault emphasizes, Kant defines the Enlightenment ‘in an almost entirely negative way: an “exit,” a “way out”’ (Foucault, 1984, p. 34) from self-imposed ‘immaturity’, which makes humans accept and stick to a given authority instead of using their own reason. Because immaturity is self-imposed, as Kant suggests, human beings are able to escape from it by a change that they will bring about themselves: by daring to know (*sapere aude*), that is, by having the courage to put one’s own reason to use (Foucault, 1984, p. 35; Kant, 2006a) – *emancipation*, even though Kant himself did not use the term (Koselleck & Grass, 1998, p. 163). Emancipation, against this backdrop, is a ‘task and obligation’ (Foucault, 1984, p. 35) moderns are confronted with *qua* being moderns, that is, *qua* being released into autonomy. The task implies the assertion of autonomy with a view to given authorities, such as God, the King, Nature (including, one may argue, Kantian takes on nature, women and races), by using their own reason. It also implies the task of limit-setting, and this is when critique comes into play.

Kant, as is well known, understood critique as the transcendental self-criticism of reason, that is, as a capacity that evaluates and corrects human knowledge and experience based on transcendental standards, which he had deemed to be accessible by reason. In the realm of morality, the categorical imperative embodies such a standard (Kant, 2006b); in the realm of politics, a republican constitution devised by a rational sovereign (Kant, 2006c). The role of critique is to distinguish between legitimate and illegitimate uses of reason. From a Kantian perspective, the use of reason is legitimate if it is beneficial to autonomy (not only to the autonomy of oneself but also that of others); and illegitimate if it ushers in ‘heteronomy, dogmatism, and illusion’ (Foucault, 1984, p. 38). Critique, as Foucault puts it, is ‘the handbook of reason that has grown up in Enlightenment; and, conversely, the Enlightenment is the age of the critique’ (Foucault, 1984, p. 38), whose ‘inheritance’ (Derrida, 1994; Allen, 2017, p. 165), one may accept as a task. Yet, one may ask, how is limit-setting still possible at a point in time in which not only God, the King and Nature have been declared dead as authoritative delimiters of freedom but also (Kantian or any other) transcendentally, trans-contextually, rationally accessible, universally valid or at least widely shared for judging the scope of and limits to autonomy? This issue – to which I will turn next – concerns both Blühdorn and Butzlaff in this Special Issue.

## Limit-setting through judgement

The idea that humans, when reasoning properly, will be led to the same conclusion is no longer credible. This has to do, as Linda Zerilli explains, with the ‘the modern descriptive fact of value pluralism…[and the]…development of modern science, which put the reliability of the senses’ and their access to transcendental truths, ‘into radical doubt’ (Zerilli, 2015, p. 2). Although no longer maintainable, the ‘idea of perfectly attuned minds following the logic of reason dies hard’ (Zerilli, 2015, p. 2). Blühdorn’s own position, which suggests that without reasonable, mature citizens, tackling the socioecological crisis is likely to become futile, appears to be a case in point (Blühdorn, in this Special Issue). What also dies hard is the idea that absent such transcendental, trans-contextual, universal criteria, widely shared and deeply entrenched criteria we run the risk of war. This fear was expressed by Hobbes in the seventeenth century who had famously argued that when people act as judges in their own case, which happens, according to Hobbes, in the pre-moral and pre-political state of nature, endless strife and the threat of death are omnipresent – hence the need for an absolute sovereign who enables and delimits freedom (Hobbes, 1996). A weakened, yet similar concern underpins Blühdorn’s account of the ‘dialectic of emancipation’ (Blühdorn, 2019, 2020a, 2020b; Blühdorn et al., 2020). This diagnosis suggests not only that late modern subjects’ claims to autonomy have become increasingly transgressive, which may lead to a ‘war of emancipation’ (Bauman, 2000, p. 51), but also that social theory and philosophy, which calls into question the very existence of ultimate standards for judgement, post-foundational thought (e.g. Laclau, 2007), may itself be complicit in spurring transgressiveness and the tipping of societies into new forms of authoritarianism.

Balancing autonomy and constraint, without which, as Kant (and modern moral, political, and legal thought more generally) stressed, autonomy is clearly endangered, remains a pressing challenge in late modern societies – a challenge in which critique continues to play a key role. The deciphering of ‘ultimate’ standards for judgement was the role Kant attributed to critique. Yet ultimate standards for judgement, including Kantian ones, do not have an altogether glorious track record in striking the right balance between freedom and constraint. They may serve as a grounding and connective tissue between people and thus contain the risk of emancipation undercutting itself, as Blühdorn suggests. Yet their ultimate, foundational nature may also lead to a declaration of war against diverging standards of judgement. Colonial rule in the name of civilization is a case in point. It is against this backdrop that post-foundational thinkers reject ultimate standards for judgement and conceive of any such standards as contingent. Yet the latter does not mean, as Blühdorn fears, that there are no foundational standards at all (Marchart, 2007). If this were the case, this would indeed mean ‘absolute freedom or total autonomy’ (Marchart, 2007, p. 14), which may easily shift towards tyranny. What the recognition of the contingency and contextual nature of criteria of judgement *does* mean is an acknowledgement of the criteria’s rootedness in the social world, in which some criteria of judgement (and related normativities) have become more institutionalized and in this sense more foundational than others.

Within this context, a late modern context that has ridden itself of ultimate foundations but not of foundations *tout court*, critique *may* and, in fact, *does* still serve as the handbook of reason that helps the latter to discern not only the scope but also the limits of autonomy. The standards critique resorts to are no longer transcendental but immanent standards – legacies of modern social, political and legal history – for distinguishing legitimate from illegitimate, right from wrong, desirable from non-desirable takes on autonomy. These legacies are operative in law no less than everyday (or non-everyday) disputes and decision. Through judgement, given scopes of and limits to autonomy are affirmed, challenged or reconfigured. In a post-foundational world, which is, to state the obvious, neither pre-moral nor pre-political, judging between legitimate and illegitimate instantiations of autonomy is no longer a matter of subsuming a particular under a transcendentally given universal by putting reason to use – an exercise that could be carried out in private – but a profoundly political activity: a ‘world building’-activity (Arendt, 1993b, pp. 219–221; Zerilli, 2016, p. 274).

Calling a given reconfiguration of emancipation illegitimate or even *perverted* or *distorted* may be, as Blühdorn suggests, an indication for a non-engagement with understandings of emancipation that differ or contradict one’s own – a non-engagement Blühdorn locates within critical social theory (Blühdorn, in this Special Issue). Yet it may also embody something else: a ‘reflective judgement’, whose nature Kant has laid out in his *Critique of Judgement* [1790] and Hannah Arendt has made fruitful for politics. A reflective judgement is the opposite of an expression of mere private preference. It implies appealing to others in order to create a ‘*sensus communis*’, the very opposite of expressing and defending merely ‘private feelings’, *sensus privates* (Arendt, 1993b, pp. 221–222). Thus when judging a given interpretation of emancipation as illegitimate and another one as legitimate, those who deliver such a judgement may be fully aware of the possibility that their drawing of boundaries is likely to evoke disagreement. They may also be aware of the fact that ‘no proof can be evoked to settle the dispute objectively’, yet they anticipate that some others will agree. Through the act of judgement, ‘one’s differences with some judging persons and one’s commonality with others’ (Zerilli, 2015, p. 5) will become manifest; through judgement, ‘political objectivity’ (Zerilli, 2015, p. 5) is established. The latter may imply a *sensus communis* that foregrounds equality as a limit to autonomy – a widespread ‘common sense’ in modernity in general and the modern history of emancipation in particular (Koselleck & Grass, 1998, p. 166), which is operative in, for instance, guaranteed social rights. It may (even at the same time) also imply a *sensus communis* that delimits autonomy by the liberation from duties to others (a liberation embodied, for instance, by ongoing pushbacks of migrants to Europe into the Mediterranean). The latter is *also* a legacy of modernity embodied by national(ist) delimitations of citizenship and citizenship rights. This begs the question of which inheritance of modernity one accepts and puts oneself at the service of, a profoundly political question, from which social theory may operate at a distance but can hardly be completely independent from.

## Conclusion

‘To articulate what is past […] means to take control of a memory, as it flashes in a moment of danger’, writes Walter Benjamin in the midst of Fascism and shortly before he took his life to escape internment (Benjamin, 2007, p. 255). Past understandings of emancipation that have foregrounded not only autonomy but also equality are clearly ‘in a moment of danger’ when established democracies find themselves on the brink of authoritarianism. They are also ‘in a moment of danger’ – or on the brink of becoming caricatures of themselves – if given realizations of emancipation undercut the long-term habitability of the planet (Crutzen et al., 2011; Rockström et al., 2009). Benjamin’s emphasis is, however, not only on ‘moments of danger’ but on how to *respond* to them. Rearticulating the past to challenge the present was his response – a response this article has endorsed with a view to emancipation.

There is no doubt that emancipation *may* devour its children: claims to autonomy may tilt towards tyranny or further entrench planetary destruction. Yet the historical meaning and function of emancipation may not be fully captured if one looks at emancipation only through the lens of processes of modernization, processes that Blühdorn depicts as a juggernaut that levels the rich and ambivalent social, political and conceptual history of emancipation, including its equally rich and ambivalent legacies. Dialectical thinking, Adorno argues, is precisely ‘the refusal to accept the denial or elimination of contradictions’ – an elimination any ‘midwife to grave-digger’ – metaphor implies. ‘Instead’, as Adorno continues, dialectical thinking ‘makes contradiction into an object or theme of […] reflection itself’ (Adorno, 2001, p. 79). Such a thinking may imply, as this article has suggested, accepting emancipation as a lingering task, fully aware of its destructive *and* transformative potentials. Accepting emancipation as a lingering task implies asserting autonomy from given social imperatives (including, potentially, the imperative of transgression) – an assertion that is the precondition for *any* critique – and the resettling of the limits to autonomy by judgement, a profoundly political activity.

Contradiction, tensions and ambivalence with a view to emancipation and given interpretations of it are alive and kicking in late modernity. They shape social constellations in which proponents of fossil-fuelled autonomy and self-determination meet proponents of post-carbon, more sustainable nature–society relations, as has been the case in recent Fridays for Future protests; in which defendants of White supremacy meet defendants of racial equality, as has been the case in the context of recent Black Lives Matter protests; in which adherents of conspiracy theories meet libertarian critics of government interventions, as has been the case in recent anti-Corona protests. One role of social theory may be that of reconstructing, and making sense of given appeals to and reformulations of emancipation. This role involves asking questions: What is at stake in a given claim to autonomy and appeal to emancipation? What is the claim’s larger context? What are the interests, normative assumptions and goals underpinning a given claim? How does a given appeal to autonomy and emancipation relate to and differ from past appeals?

Another role of social theory may be that of challenging or substantiating given appeals to and reformulations of emancipation. There are, to be sure, very good reasons for non-normative social theory (Hansen, 2016). The rapid transformation of social critique into new forms of governing led Foucault to holding his horses in respect of judging between legitimate and illegitimate takes on freedom. Yet at a point in time at which Fox News may pass as ‘truly’ critical journalism (see Lütjen, in this Special Issue); at which right-wing populist critiques of ‘political elites’ adopt the form of left-wing ones; at which White, male supremacists appropriate the language of identity politics, tracing changing notions of autonomy and emancipation may no longer suffice and *taking sides* regarding the scope and limits of given claims to autonomy and emancipation may be in order. Clearly, taking sides *is* a political activity. It is an activity through which established criteria for delimiting the scope and limits of autonomy and emancipation may be defended or new ones set. It may also be an activity through which criteria that have resonated in the past with a view to discerning critical from ideological, right from wrong, legitimate from illegitimate, and desirable from undesirable takes on autonomy may be reaffirmed ‘in a moment of danger’, and *thus* experience an afterlife in the present.
